# Supplementary material for: The EIF3H-HAX1 axis increases RAF-MEK-ERK signaling activity to promote colorectal cancer progression
Source: Nat Commun. 2024 Mar 21;15:2551. doi: 10.1038/s41467-024-46521-3 (PMC10957977; doi:10.1038/s41467-024-46521-3)
Supplement: Supplementary file 1 — Supplementary Information [file 41467_2024_46521_MOESM1_ESM.pdf]

Supplementary figure 1. EIF3H is overexpressed in Colorectal Cancer

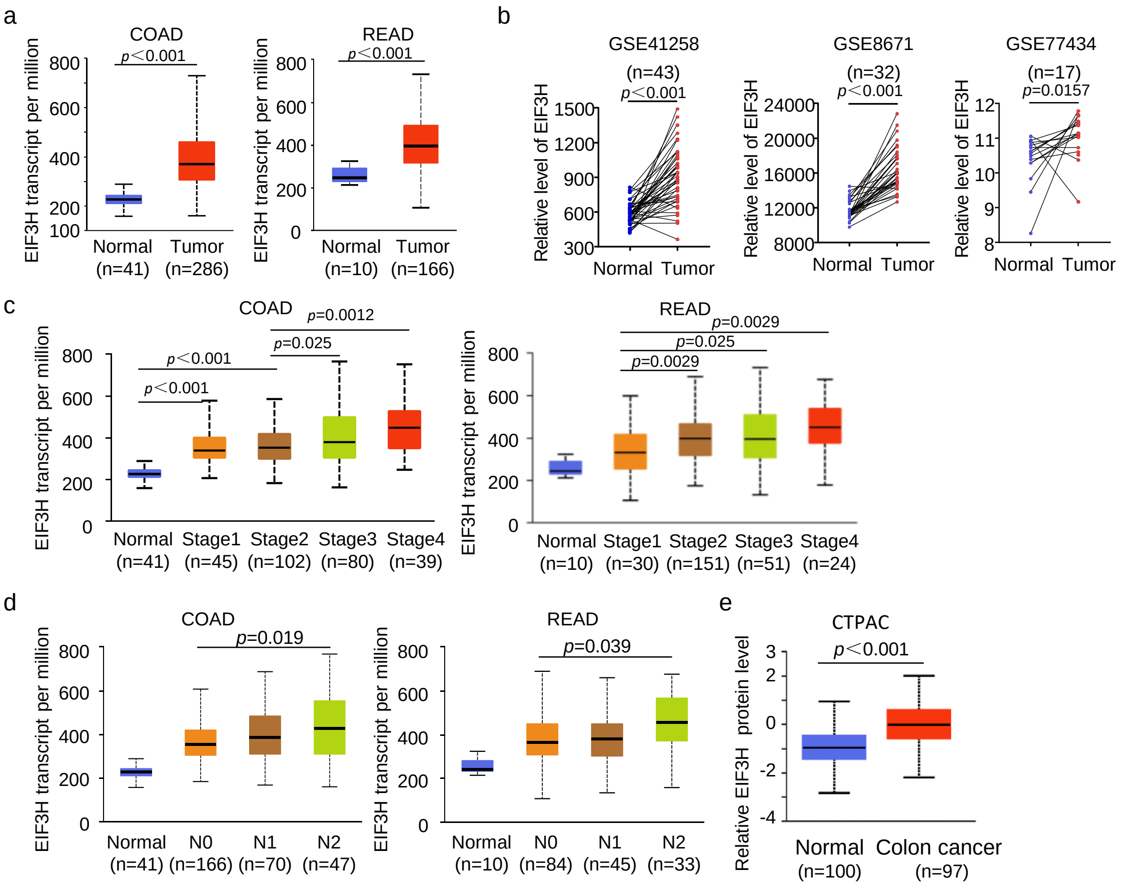

Supplementary figure 1. EIF3H is overexpressed in CRC

a Relative mRNA levels of EIF3H in the colon adenocarcinoma (COAD), rectal adenocarcinoma (READ) and normal tissues from the TCGA database. **b** Relative level of EIF3H in paired CRC and matched normal tissue samples (GSE41258, GSE8671and GSE77434). Paired student's *t* test was performed. **c** Boxplot showed expression of EIF3H in CRC based on individual cancer stages by RNA Seq–derived expression data from TCGA. **d** Boxplot showed expression of EIF3H in CRC based on nodal metastasis status by RNA Seq–derived expression data from TCGA. **e** EIF3H protein level in normal and colon cancer tissues from the Clinical Proteomic Tumor Analysis Consortium (CPTAC) database was analyzed through an online tool (http://ualcan.path.uab.edu/index.html). Z-values represent standard deviations from the median across samples for the given cancer type. Log2 Spectral count ratio values from CPTAC were first normalized within each sample profile, then normalized across samples. Data in panels a, c, d and e are presented as boxplot (the horizontal lines in the boxplots represent the median, the boxes represent 25th and 75th percentiles and the whiskers represent the min and max values). The *p* values were determined by unpaired two-tailed *t* test for panels a, c, d and e. Source data are provided as a Source Data file.

Supplementary figure 2. Transgenic mice study.

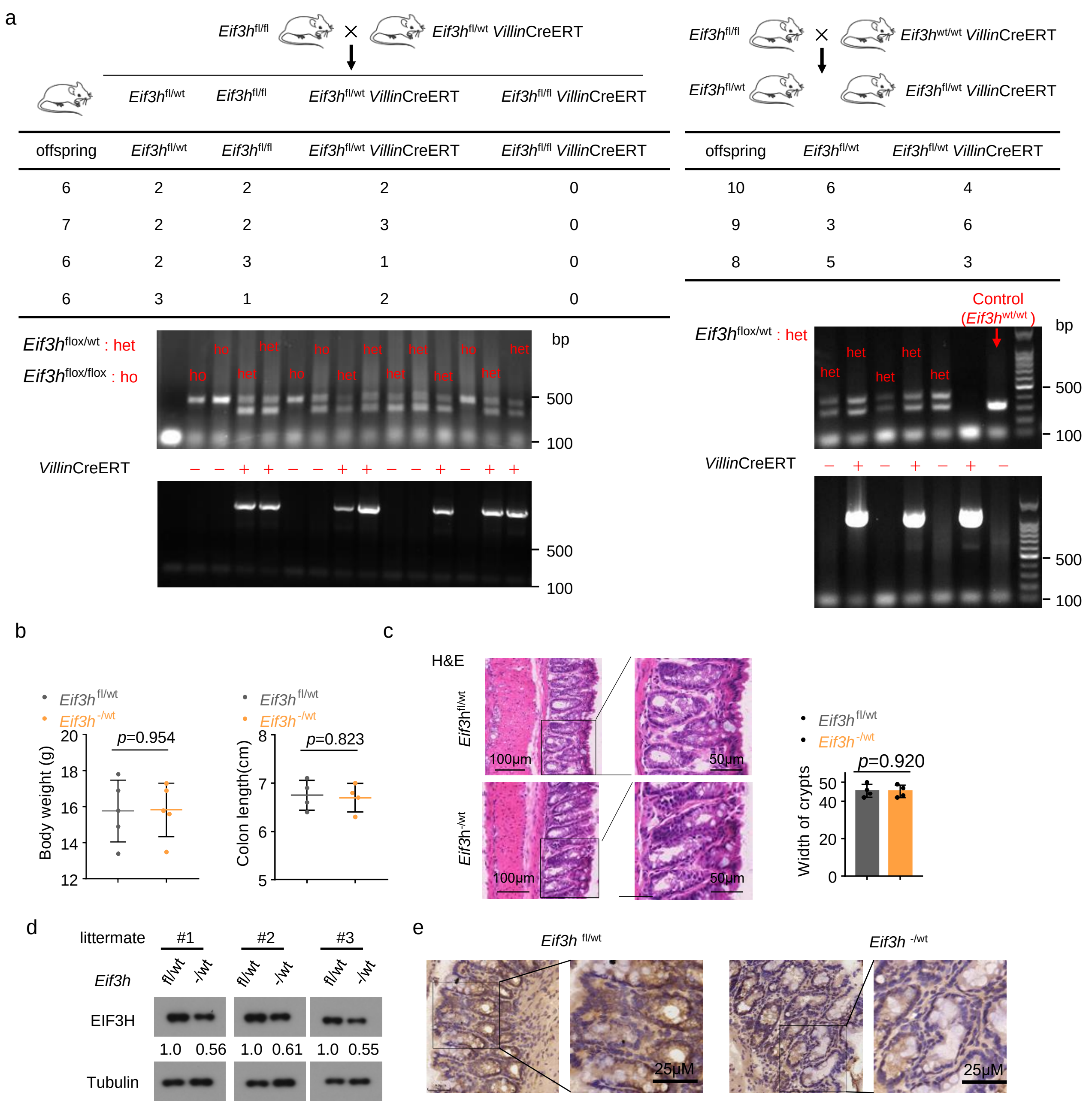

offspring	Eif3h fl/wt	Eif3h fl/fl	Eif3h fl/wt VillinCreERT	Eif3h fl/fl VillinCreERT
6	2	2	2	0
7	2	2	3	0
6	2	3	1	0
6	3	1	2	0

offspring	Eif3h fl/wt	Eif3h fl/wt VillinCreERT
10	6	4
9	3	6
8	5	3

Supplementary figure 2. Transgenic mice study.

a Breeding records and PCR analysis for *Eif3h*$^{flox/wt}$ and *Eif3h*$^{flox/wt}$, *Villin*-CreERT mice. **b** Mice body weight and colon length of littermate *Eif3h*$^{flox/wt}$ and *Eif3h*$^{flox/wt}$, *Villin*-CreERT mice treated with tamoxifen. **c** H&E staining for colon tissues obtained from the indicated tamoxifen-induced mice. The colon was analyzed for the width of crypts from 2 male and 2 female mice in a total of 50 crypts per genotype. **d** Protein levels of Eif3h in indicated colon tissues. **e** Representative images of Immunohistochemistry staining of colon tissues obtained from the indicated tamoxifen-induced mice. Scale bar = 25μm. Data are presented as the means ± SD. The *p* values were obtained by two-tailed unpaired *t* test. Source data are provided as a Source Data file.

Supplementary Figure 3. EIF3H knockdown inhibits CRC cell growth, migration and invasion

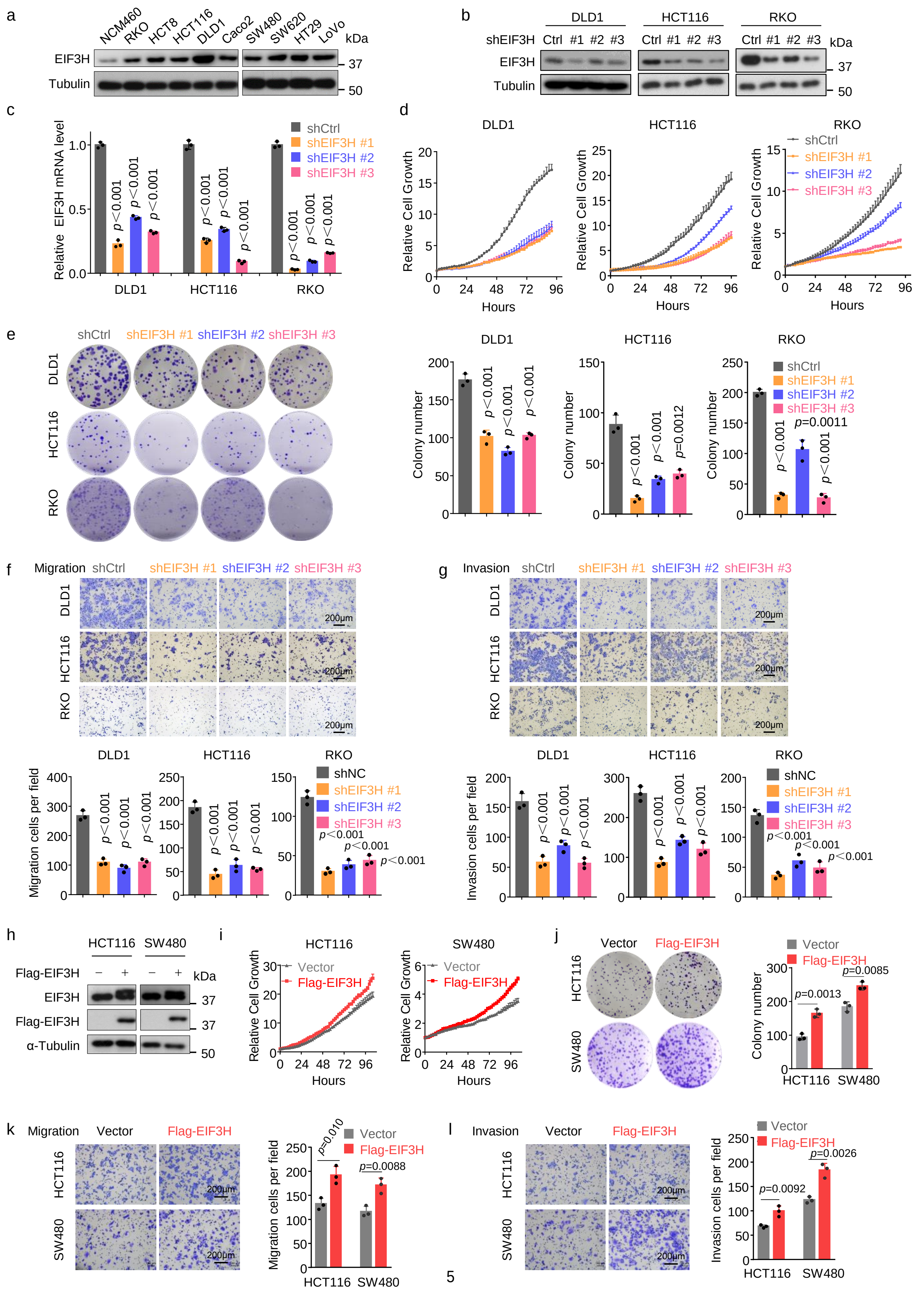

Supplementary Figure 3. EIF3H knockdown inhibits CRC cell growth, migration and invasion

a Levels of EIF3H proteins in a normal colon epithelial cell line NCM460 and nine human CRC cell lines. α-Tubulin was used as a loading control. **b, c** Knockdown efficiency of EIF3H in DLD1, HCT116 and RKO cells was verified by western blotting and qRT-PCR. **d, e** The cell proliferation and colony formation of EIF3H-knockdown CRC cells were measured by Incucyte assays, colony formation assays. **f, g** The motility of EIF3H-knockdown CRC cells was assessed by transwell migration and matrigel invasion assays.

h-l Overexpression of EIF3H in HCT116 and SW480 cells was verified by western blotting. The cell proliferation, colony formation and motility were detected. The number of migrated or invaded cells was shown in the bar chart. The values represented the mean ± SD of 3 independent experiments. The *p* values were obtained by two-tailed unpaired t test (**c, e-g, j, k, l**), Scale bar = 200μm. Representative immunoblots shown in figures were repeated three times independently with similar results. Source data are provided as a Source Data file.

Supplementary Figure 4. Knockdown EIF3H decreases HAX1 protein level in CRC cells

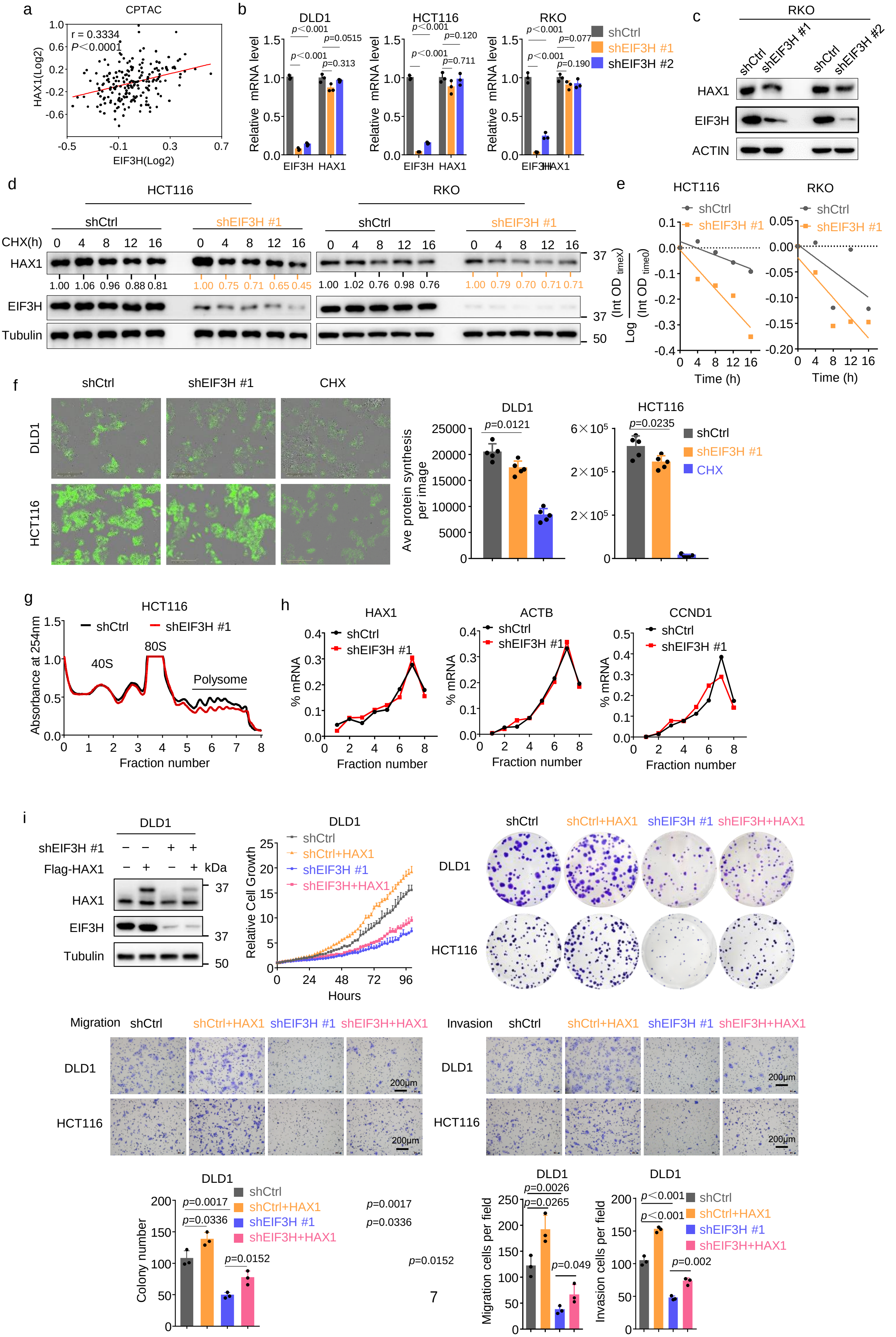

Supplementary Figure 4. Knockdown EIF3H decreases HAX1 protein level in CRC cells

a Spearman rank correlation analysis of EIF3H and HAX1 protein expression in CRC tumors from CPTAC colon cancer cohort. **b, c** qRT-PCR and western blot analysis of HAX1 level in shCtrl and shEIF3H DLD1, HCT116 and RKO cells. **d, e** HAX1 turnover rate was analyzed by CHX pulse-chase assay in HCT116 and RKO cells. **f** Evaluation of EIF3H knockdown on nascent protein synthesis. Two days after doxycycline induction, O-propargyl-puromycin (OPP) was added to the culture medium to label nascent peptides which were visualized by Immunofluorescence microscope after fixation with fluorescent Click iT chemistry. The fluorescence intensity of all the cells corresponded to protein synthesis. Representative images of protein synthesis were shown. Data represent means ± SD from five independent experiments. **g, h** Polysome profiles of EIF3H KD and control cells. The EIF3H-knockdown HCT116 cells were lysed and subjected to sucrose gradient centrifugation. The sucrose gradient profiles were obtained by continuous scanning at A254. The positions in the gradients of 40S subunits, 80S ribosomes, and polysomes were labeled (**g**). The translational status of specific mRNA in EIF3H KD and control cells were examined by qRT-PCR (**h**). **i** EIF3H knockdown DLD1 and HCT116 cells were infected with lentivirus containing control vector or Flag-HAX1 vector. The indicated protein level, cell proliferation, colony formation and motilities were detected. Data are presented as the means ± SD, n = 3 biologically independent experiments. The *p* values were obtained by two-tailed unpaired *t* test for panel b, f and i. Scale bar = 200μm. Representative immunoblots shown in figures were repeated three times independently with similar results. Source data are provided as a Source Data file.

Supplementary Figure 5. Loss-of-function EIF3H mutations attenuate its effect on stabilizing HAX1

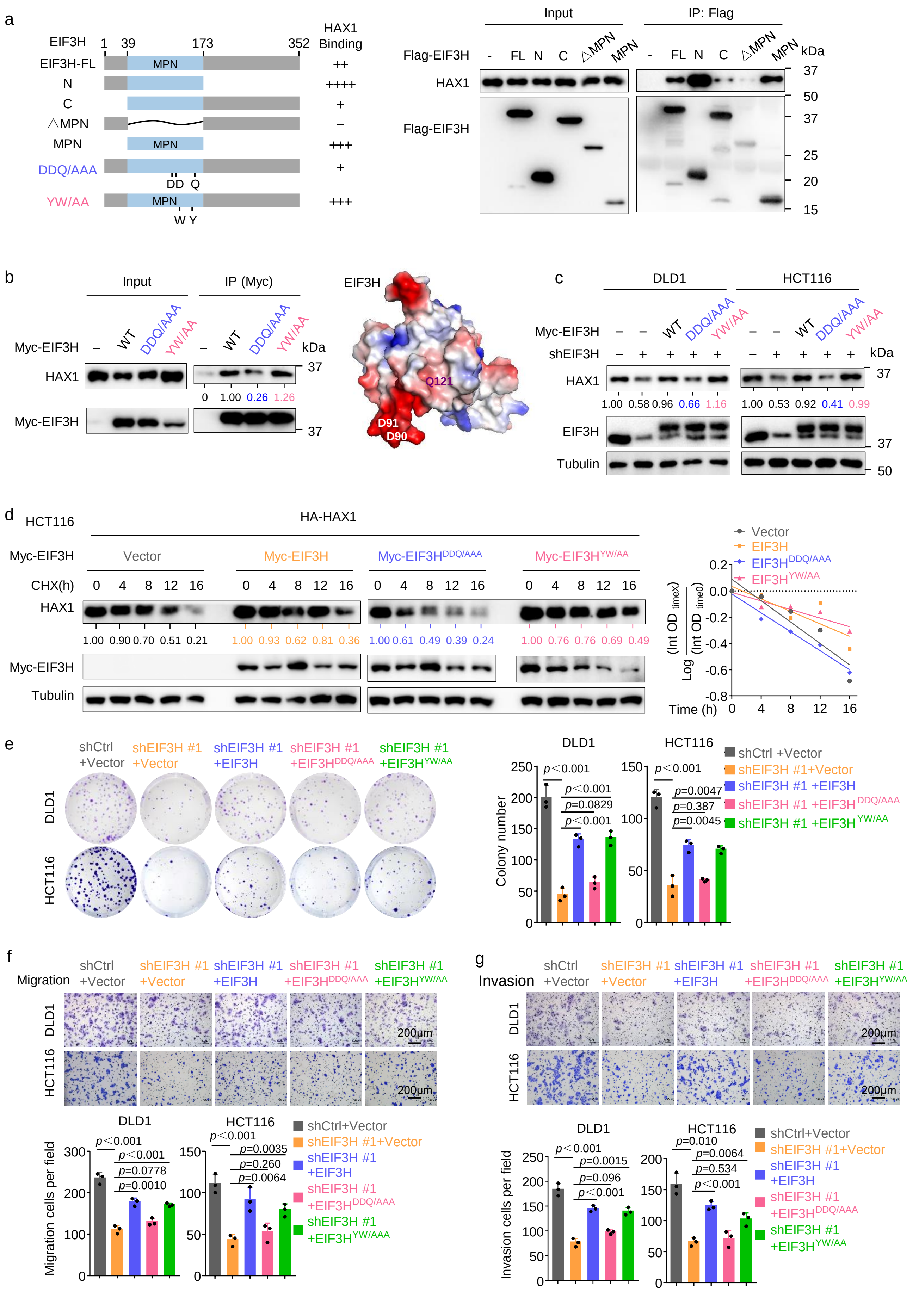

Supplementary Figure 5. Loss-of-function EIF3H mutations attenuate its effect on stabilizing HAX1

a Flag tagged EIF3H of full length wild-type, N-terminal (1-173aa), C-terminal (39-354aa), △MPN(1-39+174-354) and MPN(39-173) was transfected into 293T cells. Cell lysates were immunoprecipitated with anti-Flag M2 beads and immunoblotted with indicated antibodies for binding studies. **b** Myc tagged EIF3H of full length wild-type, DDQ-AAA dot mutant EIF3H and YW-AA mutant dot EIF3H was transfected into 293T cells. Cell lysates were immunoprecipitated with anti-Myc beads and immunoblotted with indicated antibodies for binding studies. **c** Western blot analysis of protein expression of HAX1 in in DLD1 and HCT116 cells transfected with the indicated shRNA and plasmids targeted EIF3H.

d Representative immunoblots showing the turnover rate of HA-tagged HAX1, with or without Myc-tagged EIF3H or dot mutant EIF3H overexpression in HCT116 cells (left). Transfected HCT116 cells were treated with CHX (100 μg/mL) for the indicated times. Quantification of HAX1 turnover rate by the Image J software (right). **e-g** EIF3H knockdown DLD1 and HCT116 cells were transfected with wild type and dot mutant EIF3H. The cell colony formation (**e**), transwell migration (**f**) and invasion (**g**) ability were detected. Data are presented as the means ± SD, n = 3 biologically independent experiments. The *p* values were obtained by two-tailed unpaired *t* test. Scale bar = 200μm. Representative immunoblots shown in figures were repeated three times independently with similar results. Source data are provided as a Source Data file.

Supplementary Figure 6. The half-life of HAX1 is increased in cells transfected with βTrCP specific siRNA.

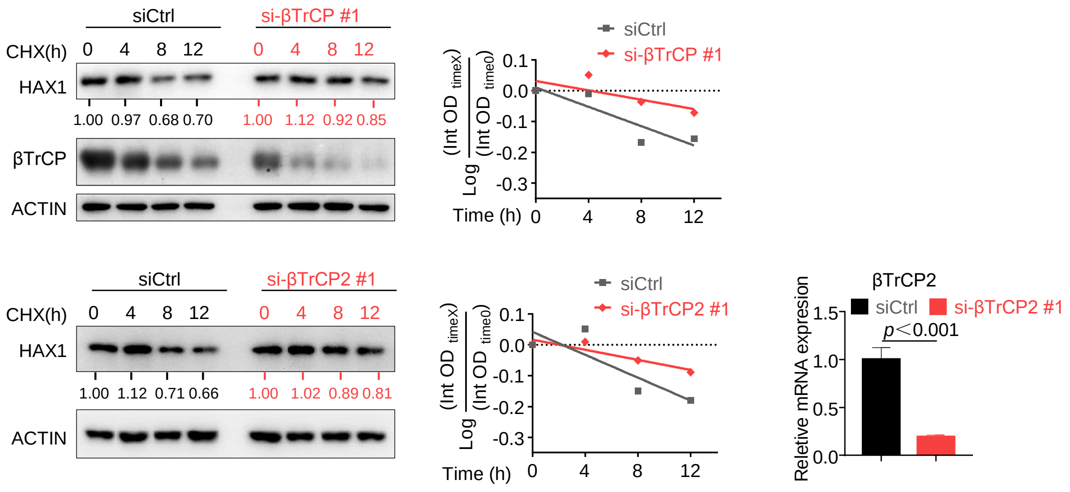

Supplementary Figure 6. The half-life of HAX1 is increased in cells transfected with βTrCP specific siRNA. HAX1 turnover rate was analyzed by CHX assay in HCT116 cells transfected with siRNA targeted βTrCP or βTrCP2. Data are presented as the means ± SD, n = 3 biologically independent experiments. The *p* values were determined by unpaired two-tailed *t* test. Representative immunoblots shown in figures were repeated three times independently with similar results. Source data are provided as a Source Data file.

Supplementary Figure 7

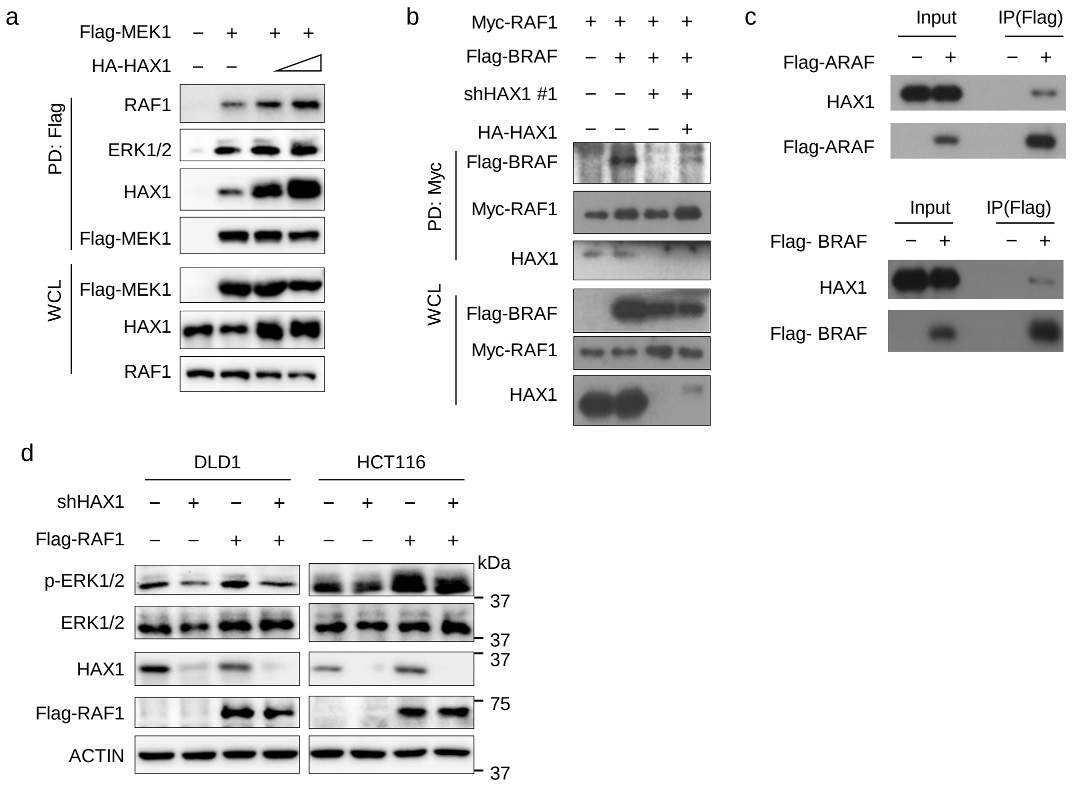

Supplementary Figure 7. HAX1 enhances the interaction between RAF1, MEK1 and ERK1, thereby potentiating phosphorylation/activation of ERK1/2

a The HEK293T cells were transfected with Flag-MEK1 with increasing HA-HAX1 plasmid. Cell lysates were immunoprecipitated with anti-Flag M2 beads and immunoblotted with indicated antibodies. WCL, whole cell lysis. **b** The shCtrl and shHAX1 HCT116 cells were transfected with Myc-RAF1, Flag-BRAF with or without HA-HAX1 plasmids. Cell lysates were immunoprecipitated with anti-Myc beads and immunoblotted with indicated antibodies. **c** HEK293T cells were transfected with Flag-ARAF (top) or Flag-BRAF (bottom) plasmids. Cell lysates were immunoprecipitated with anti-Flag M2 beads and immunoblotted with indicated antibodies. **d** The shCtrl and shHAX1 CRC cells were transfected with Flag-RAF1 plasmids. Western blot analysis of indicated proteins. Representative immunoblots shown in figures were repeated three times independently with similar results. Source data are provided as a Source Data file.

Supplementary Figure 8

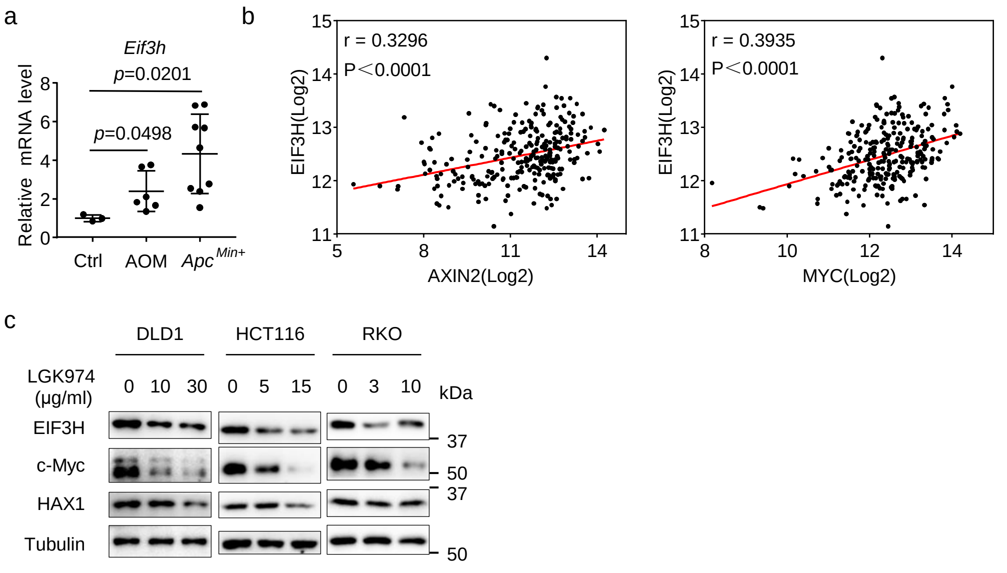

Supplementary Figure 8. Activation of Wnt/β-catenin signaling induces EIF3H expression in CRC cells

a Using GEO2R of PubMed (http://www.ncbi.nlm.nih.gov/geo/geo2r/), we assessed changes in *Eif3h* mRNA levels in Gene Expression Omnibus datasets of colon tumors isolated from $Apc^{Min/+}$ and AOM-treated mice (GSE5204). The *p* values were determined by unpaired two-tailed *t* test. **b** Spearman rank correlation analysis of EIF3H and AXIN2, MYC mRNA level in CRC tumors from TCGA-CRC cohort. **c** Western-blot analysis of EIF3H and c-Myc level in DLD1, HCT16 and RKO cells treated with different concentrations of Wnt pathway inhibitor LGK974. Representative immunoblots shown in figures were repeated three times independently with similar results. Source data are provided as a Source Data file.

Supplementary table 1. Sequence of some βTrCP-recognized degron.

β-TrCP substrates	Degron
IκBα	DSGLDS
Snail	DSGKGS
ATF4	DSGICMS
WEE1	DSAFQE
p100	DSAYGS
CDC25A	STDSG
MCL1	DGSLPS
TIPE2	MESFSSKS

Supplementary table 2. Association between EIF3H expression and clinicopathological features in 104 CRC cases.

Features	Total	Low EIF3H	High EIF3H	*p* value
Tumor size				0.170
<5	43	21	18	
≥5	65	26	39	
Histological grade				0.101
I - II	6	4	2	
II	51	25	26	
II - III	34	10	24	
III	13	8	5	
pT status				0.607
T1	1	0	1	
T2	5	3	2	
T3	81	39	42	
T4	17	5	12	
pN status				0.099
N0	64	33	31	
N1+N2	40	14	26	
pM status				0.111
M0	100	47	54	
M1	3	0	3	
Clinical stage				0.140
I+ II	65	33	32	
III+ IV	39	14	25	

The *p* values were calculated in SPSS19 using a chi-square test. All *p* values were two sided and the level of statistical significance was set at < 0.05.

Supplementary table 3. Cox proportional hazard regression analyses of potential poor prognostic factors in colorectal cancer.

Clinicopathological features	Univariate analysis		Multivariate analysis	
	HR (95% CI)	*p* value	HR (95% CI)	*p* value
EIF3H expression (low vs. high)	1.72(1.03-2.87)	0.040	1.28(0.74-2.22)	0.384
Tumor size (<5 cm vs. ≥5 cm)	1.22(0.73-2.04)	0.443	1.38(0.79-2.42)	0.254
Differentiation (High/moderate vs. low)	1.81(1.10-2.98)	0.018	1.30(0.76-2.227)	0.343
pT status (T1+T2 vs. T3+T4)	2.53(0.62-10.34)	0.195	1.829(0.43-7.80)	0.418
pN status (N0 vs. N1+N2)	2.94(1.78-4.86)	<.001	2.77(1.59-4.83)	<.001
pM status (M0 vs. M1)	14.86(4.14-53.30)	<.001	6.64(1.80-24.58)	0.005

Hazard ratios (HRs), 95% confidence intervals (CIs) and *p* values were calculated using univariate or multivariate Cox proportional hazards regression in SPSS 19. All *p* values were two sided and the level of statistical significance was set at < 0.05.

Supplementary table 4. Detailed information of sequences of shRNA and siRNA used in this study.

Gene	Sequence (5' -3')
EIF3H shRNA #1	GCAACTCTTGGAAGAAATATA
EIF3H shRNA #2	CCCAAGGATCTCTCTCACTAA
EIF3H shRNA #3	CGCCATGTAAACATTGATCAT
HAX1 shRNA #1	ACAGACACTTCGGGACTCAAT
HAX1 shRNA #2	CCAAATCCTATTTCAAGAGCA
HAX1 shRNA #3	ACTCGAGATGAAGATGATGAT
βTrCP1 siRNA #1	GCAGAGAGATTTCATAACT
βTrCP2 siRNA #1	CTGCGACCGACATCACTTT

Supplementary table 5. Primer sequences for qRT-PCR used in this study.

Gene	Forward sequence	Reverse sequence
EIF3H	CAGATGGAAATGATGCGGAGC	AGTATGTGGACTGATACCAGCC
HAX1	AGCATCTTCAGCGATATGGGG	CCTCCCGTAGTCTCTCACCA
cMyc	GTCAAGAGGCGAACACACAAC	TTGGACGGACAGGATGTATGC
ACTB	CATGTACGTTGCTATCCAGGC	CTCCTTAATGTCACGCACGAT
AXIN2	TACACTCCTTATTGGGCGATCA	TTGGCTACTCGTAAAGTTTTGGT
CCND1	CAATGACCCCGCACGATTTC	CATGGAGGGCGGATTGGAA
βTrCP2	CCAAGGTCTTTGTGGCTAGGC	GGCCTCTTTCTGGAGACGATC

Supplementary table 6. The primers specific for EIF3H promoter used in this study.

	Sequence (5' -3')
EIF3H promoter -1F	ATTTTGAGTGTATCTCTTTGT
EIF3H promoter -1R	TGGCTACCTCCTTTATTCTCA
EIF3H promoter -2F	TAGTGTATTTCATAAAGAGGG
EIF3H promoter -2R	CCTCATAGCAGTAAAACTACAA
EIF3H promoter -3F	TCCAGCTACCTAGTGGTAGGC
EIF3H promoter -3R	CCAAACCCTAGGTCACACAGA
EIF3H promoter -4F	CTAGGGTTTGGTGTATTTTAA
EIF3H promoter -4R	AATTAAATCAATAAACGGCAT
EIF3H promoter -5F	CCGTTTATTGATTTAATTATTT
EIF3H promoter -5R	ACATCTCAAGTCCTTAAAAGA
EIF3H promoter -6F	TATGCATAATGCGAGTTAGTA
EIF3H promoter -6R	CTTTGTGCATGCCTACTGTAC
