# Supplementary material for: The EIF3H-HAX1 axis increases RAF-MEK-ERK signaling activity to promote colorectal cancer progression
Source: Nat Commun. 2024 Mar 21;15:2551. doi: 10.1038/s41467-024-46521-3 (PMC10957977; doi:10.1038/s41467-024-46521-3)
Supplement: Supplementary file 2 — Description of Additional Supplementary Files [file 41467_2024_46521_MOESM2_ESM.pdf]

Description of Additional Supplementary Files

File name: **Supplementary Data 1**

Description: The pathological feature and EIF3H expression score of the human colorectal cancer tissue array containing 104 CRC cases. The EIF3H expression score was based on immunohistochemistry staining in tumor tissues.
